# *Anaplasma phagocytophilum* ecotype distribution and zoonotic potential in questing and feeding *Ixodes ricinus* from Germany

**DOI:** 10.1016/j.crpvbd.2025.100339

**Published:** 2025-11-27

**Authors:** Andrea Springer, Daniela Angulo Mora, Daniela Jordan, Christina Strube

**Affiliations:** Institute for Parasitology, Centre for Infection Medicine, University of Veterinary Medicine Hannover, Buenteweg 17, 30559, Hanover, Germany

**Keywords:** Tick-borne disease, Zoonosis, Granulocytic anaplasmosis, HGA, Human, Dog, Cat

## Abstract

*Anaplasma phagocytophilum* is a genetically diverse tick-borne pathogen, subdivided into different phylogenetic clusters, so-called ecotypes, with distinct transmission cycles. European variants pathogenic for humans, dogs, cats and horses are transmitted by *Ixodes ricinus* and belong to a distinct subgroup within Ecotype I. Ecotype II is also transmitted by *I. ricinus*, but considered primarily adapted to roe deer. The present study investigated the *A. phagocytophilum* ecotype distribution in questing and feeding *I. ricinus* from Germany, based on sequence analysis of the partial *groEL* gene. Of 94 positive questing ticks (34 nymphs, 60 adults) from the city of Hanover, 90.4% harboured Ecotype I and 9.6% Ecotype II, with 78.7% of sequences clustering within the zoonotic subgroup. The proportion of Ecotype I was significantly lower in feeding ticks collected countrywide. Among 27 ticks (10 nymphs, 17 adults) from humans, the Ecotype I/Ecotype II ratio was 55.6%/44.4%, with potentially zoonotic isolates in 40.7% of ticks. From dogs and cats, 57 and 28 positive female ticks yielded *groEL* sequences, respectively, with an Ecotype I/Ecotype II ratio of 68.4%/31.6% in ticks from dogs, and 60.7%/39.3% in ticks from cats. Potentially zoonotic isolates were detected in 54.4% of positive ticks from dogs, and 14.3% from cats. The differences in ecotype distribution may be due to geographical differences, variation between urban and rural habitats, and differences in host behaviour. The high proportion of isolates potentially pathogenic to humans and pets highlights the importance of preventive measures to avoid tick infestation.

## Highlights

• *Anaplasma phagocytophilum groEL* ecotypes assessed in questing and feeding *Ixodes ricinus.*
• Higher Ecotype I proportion was found in questing ticks from an urban area than in feeding ticks.
• The frequency of potentially zoonotic isolates (78.7%) was greatest in urban questing ticks.
• Ecotype distribution may be shaped by rural-urban differences and host behaviour.
• Significantly higher *msp2/p44* copy numbers of Ecotype II than Ecotype I were found.

## Graphical abstract

**Figure:**
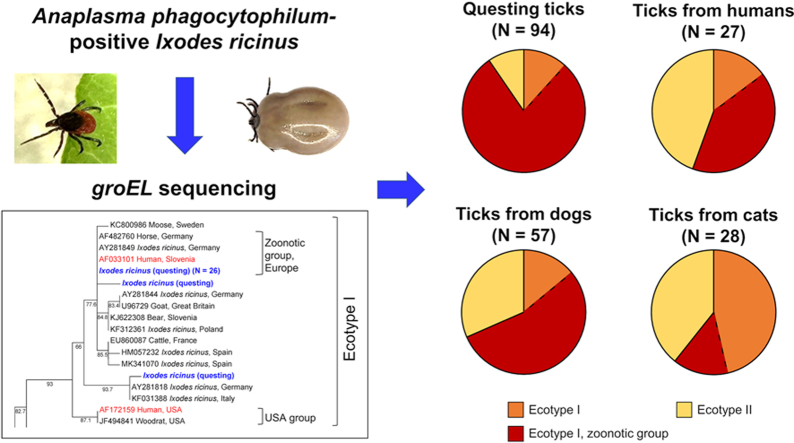

## 1 Introduction

*Anaplasma phagocytophilum* is a zoonotic tick-borne pathogen with a wide host range, causing granulocytic anaplasmosis in humans, dogs, cats, horses and domestic ruminants (Dugat et al., 2015). Clinical cases are associated with fever, thrombocytopenia, anaemia and immunosuppression, among others (Dumler et al., 2005). Different *Ixodes* spp. are recognized as vectors (Macleod and Gordon, 1933; Telford et al., 1996; Des Vignes et al., 1999; Teglas and Foley, 2006), whereby the widespread castor bean tick *Ixodes ricinus* is of principal importance in Europe (Matei et al., 2019).

While cases of human granulocytic anaplasmosis (HGA) are common in North America (Bakken and Dumler, 2015; Dahlgren et al., 2015), the incidence in Europe is much lower (Matei et al., 2019; Azagi et al., 2020). Nevertheless, presence of antibodies against *A. phagocytophilum* indicates frequent exposure of the population, e.g. in 8.1% of professionally tick-exposed workers and 6.2% of rural blood donors in Belgium (De Keukeleire et al., 2017). An even higher seropositivity rate of 17.7% was determined in forestry workers in Poland (Cisak et al., 2005). In dogs, granulocytic anaplasmosis is regarded as one of the most important vector-borne diseases in central and northern Europe. Nevertheless, high seroprevalence rates, e.g. of more than 30% in certain federal states of Germany (Krupka et al., 2007; Schäfer et al., 2023), indicate that many canine infections are probably subclinical or not diagnosed due to unspecific symptoms.

*Anaplasma phagocytophilum* is characterized by a high genetic diversity (Rar et al., 2021), and the difference in HGA incidence between North America and Europe has been attributed to a different prevalence of strains with zoonotic potential (Matei et al., 2019; Aardema, 2023). Based primarily on the *groEL* heat-shock operon gene, different ecotypes with distinct vector and host associations have been identified (Jahfari et al., 2014; Jaarsma et al., 2019). These patterns are also supported by multi-locus sequence typing and *ankA* gene phylogenies (Rar et al., 2021). Most human-derived isolates cluster within *groEL* Ecotype I, which is transmitted by *I. ricinus* and has the broadest vertebrate host range (Jahfari et al., 2014; Rar et al., 2021). Strains causing disease in dogs, cats and horses also belong to this ecotype (Dondi et al., 2014; Hovius et al., 2018; Rar et al., 2021; Kruppenbacher et al., 2023). Indeed, zoonotic strains from Europe form a monophyletic group within Ecotype I, together with almost all isolates from horses, dogs, cats, wild boars, red foxes, and hedgehogs (Lesiczka et al., 2025), whereas the remaining vertebrate isolates in Ecotype I were primarily derived from red deer, sheep, goats and chamois (Rar et al., 2021). Ecotype II is also transmitted by *I. ricinus*, but is considered a host-specialist, primarily linked to roe deer in Europe (Jahfari et al., 2014; Rar et al., 2021). Ecotype III seems to form a separate enzootic cycle involving voles, shrews, and the rodent-tick *Ixodes trianguliceps*, while Ecotype IV is primarily associated with birds (Jahfari et al., 2014; Rar et al., 2021; Rouxel et al., 2024). In addition, another rare ecotype was described from *Ixodes ventalloi* in Spain (Santos et al., 2018).

Multiple studies have investigated the prevalence of *A. phagocytophilum* in questing *I. ricinus*, with values below 10% in most European regions (Rosef et al., 2009; Matei et al., 2015; Knoll et al., 2021; Gandy et al., 2022). In Germany, local prevalence rates of up to 14.8% have been determined (Knoll et al., 2021), but data on the ecotype distribution in questing ticks are limited to a single study (Hamšíková et al., 2019). As knowledge on the frequency of potentially pathogenic strains is important to accurately assess the risk for humans and domestic animals, the aim of the present study was to investigate the ecotype distribution in *A. phagocytophilum*-positive questing *I. ricinus* as well as in specimens removed from humans, dogs and cats from Germany.

## 2 Materials and methods

### 2.1 Available *A. phagocytophilum*-positive questing ticks

Questing ticks were collected during the years 2015 and 2020 at ten locations in the city of Hannover, Germany, morphologically identified to the species level (Estrada-Peña et al., 2004, 2017), subjected to DNA isolation and tested individually for *A. phagocytophilum* infection by probe-based qPCR targeting the *msp2/p44* gene in the frame of previous studies (Blazejak et al., 2017; Glass et al., 2022). In each year, 2100 ticks were investigated, resulting in 79 qPCR-positive samples (3.8%) from 2015 (Blazejak et al., 2017) and 63 (3.0%) from 2020 (Glass et al., 2022). Of these, 75 (37 adults and 38 nymphs) and 50 (45 adults and 5 nymphs) were available for ecotype differentiation, respectively.

### 2.2 Available *A. phagocytophilum*-positive ticks from dogs and cats

Feeding ticks from dogs and cats were collected in veterinary practices across Germany during 2020–2021 (Probst et al., 2024). Pet owners of the sampled animals provided background information, including whether the animal lived in a rural or urban area, *via* a questionnaire (Probst et al., 2025). After morphological species identification (Estrada-Peña et al., 2017), 1500 *I. ricinus* females of different engorgement states were individually tested from each host species by *msp2/p44* probe-based qPCR, with *A. phagocytophilum* detection frequencies of 19.0% (285/1500) in ticks from dogs and 30.9% (464/1500) in ticks from cats (Probst et al., 2024). From this study, 266 qPCR-positive samples from dogs and 377 from cats were subjected to ecotype differentiation.

### 2.3 Ticks from humans

Ticks from humans in different states of engorgement were sent as diagnostic material to the Institute for Parasitology, University of Veterinary Medicine Hannover, Germany, during 2015–2023. After species identification based on morphological criteria (Estrada-Peña et al., 2004, 2017), individual ticks were homogenized and subjected to DNA isolation using the NucleoSpin 8 Blood Kit (Macherey-Nagel, Düren, Germany) as described previously (Tappe and Strube, 2013; May and Strube, 2014), or the blackPREP Tick DNA/RNA kit (Analytik Jena AG, Jena, Germany) according to the manufacturer’s instructions. As of mid-2019, the blackPREP Tick DNA/RNA kit was used exclusively. In addition to 955 *I. ricinus* specimens for which *A. phagocytophilum* testing was requested as a diagnostic service between 2015 and 2023, all additional *I. ricinus* ticks received during the year 2020 for detection of other pathogens (*n* = 386) were additionally tested for *A. phagocytophilum* in the present study. In routine diagnostic testing until mid-2023, 10 μl DNA template was used in a 25 μl qPCR reaction targeting the *msp2/p44* gene based on a primer-probe combination by Courtney et al. (2004), with reaction set-ups and thermal cycling conditions as described by Tappe et al. (2014). Thereafter, the template amount was reduced to 2 μl to further increase sensitivity, as inhibitory components in feeding ticks might interfere with the qPCR reaction (Probst et al., 2024). All samples from 2020 were (re-)tested with 2 μl template, including 143 samples which had previously been evaluated based on 10 μl template. All reactions were run in duplicate and included a no-template control. A serial plasmid standard (10^0^–10^6^ copies) for quantification was also included for ticks from 2020, while only a positive control (10^4^ copies) was included for routine diagnostic testing.

### 2.4 Amplification of the partial *groEL* gene

Samples with a positive qPCR result were subjected to (nested) conventional PCR targeting the *groEL* gene. At first, primers EphplgroEL(569)F and EphgroEL(1142)R (Alberti et al., 2005) were used to amplify a 574-bp fragment. In case no or only a weak band was observed, a second round with primers ApNest-F and ApNest-R (Jaarsma et al., 2019) was performed to amplify a 407-bp fragment. The 25 μl reaction set-up included 0.25 μl DreamTaq polymerase (5 U/μl, Thermo Fisher Scientific Inc., Schwerte, Germany), 2.5 μl 10× buffer, 0.5 μl of each primer (10 μM each), 0.5 μl dNTPs (10 mM each, Roti®-Mix PCR 3, Carl Roth, Karlsruhe, Germany) and 10 μl template. The thermoprofile included 95 °C for 3 min, 40 cycles of 94 °C for 30 s, 56 °C for 30 s, and 72 °C for 60 s, followed by final elongation at 72 °C for 10 min. In the second round, 1 μl of PCR product from the first round was used as template and the annealing temperature was adjusted to 55 °C. After visualization on GelRed® (Biotium Inc., Fremont, CA, USA) supplemented 1.5% agarose gels, PCR products were custom Sanger sequenced (Microsynth Seqlab GmbH, Göttingen, Germany).

### 2.5 Phylogenetic analyses

After quality control and trimming of primer sequences, the obtained sequences were aligned with publicly available sequences obtained from NCBI GenBank in Clone Manager Professional v. 9 (Sci Ed Software, Westminster, Colorado, USA). Representative sequences of *A. phagocytophilum* ecotypes I-IV were chosen based on the *groEL* phylogenetic tree presented by Rar et al. (2021). Alignments of each sample set were trimmed to a common length: 291 nucleotide sites for questing ticks, 338 for ticks from humans, 292 for ticks from dogs and 290 for ticks from cats. Maximum likelihood phylogenetic trees were computed *via* the IQ-TREE web server (Trifinopoulos et al., 2016), which uses ModelFinder to choose the best-fit substitution model according to the Bayesian information criterion (BIC) (Kalyaanamoorthy et al., 2017). Branch support values were estimated based on ultrafast bootstrap with 1000 replicates (Hoang et al., 2017).

### 2.6 Statistical analyses

Statistical analyses were carried out in R v. 4.4.0 (R Core Team, 2024). The *A. phagocytophilum* detection frequency was compared between nymphal and adult ticks from humans *via χ*^2^-test. Differences in ecotype distribution were assessed between tick developmental stages, between the different years for questing ticks, and between the total questing ticks and ticks derived from humans, cats, and dogs using Fisher’s exact test, followed by Bonferroni-Holm correction of *P*-values in case of multiple comparisons. In addition, the influence of a rural or urban residence on the ecotype distribution in ticks from dogs and cats was investigated using a binomial generalized linear model (GLM), with host species as a fixed factor in addition to residence type.

The *msp2/p44* copy numbers were compared between nymphal and adult ticks *via* Wilcoxon rank sum test. To investigate possible differences in *msp2/p44* copy numbers between *A. phagocytophilum* ecotypes while taking tick stage into account, a linear mixed model (LMM) was fitted for log-transformed copy numbers, including the origin of the sample (questing 2015, questing 2020, human-, dog- or cat-associated) as a random factor.

Models were compared to null models containing only the intercept (GLM) or the random factor (LMM), respectively, in a likelihood ratio test.

## 3 Results

### 3.1 *Anaplasma phagocytophilum* ecotyping

Overall, amplification and sequencing were successful for 206 of the 810 *A. phagocytophilum*-positive tick samples. Of the samples that yielded no result, the majority produced no amplicon, while only approximately 10% yielded either no sequence, a non-*A. phagocytophilum* sequence (mostly *Pseudomonas* sp.) or had to be discarded due to a low sequence quality. The overall success depended on *msp2/p44* copy numbers. Among the 785 ticks with determined copy numbers, the success rate amounted to 70.5% (62/88) for samples containing ≥ 10^4^ copies, 53.3% (32/60) for samples with ≥ 10^3^ to < 10^4^ copies, 32.6% (14/43) for samples with ≥ 10^2^ to < 10^3^ copies, 13.2% (20/152) for samples with ≥ 10^1^ to < 10^2^ copies, and 14.3% (63/442) for those with < 10^1^ copies. Of the successfully differentiated samples, 75.7% (156/206) were assigned to *A. phagocytophilum* Ecotype I and 24.3% (50/206) to Ecotype II.

### 3.2 *Anaplasma phagocytophilum* ecotypes in nymphal and adult ticks

**Fig. 1:**
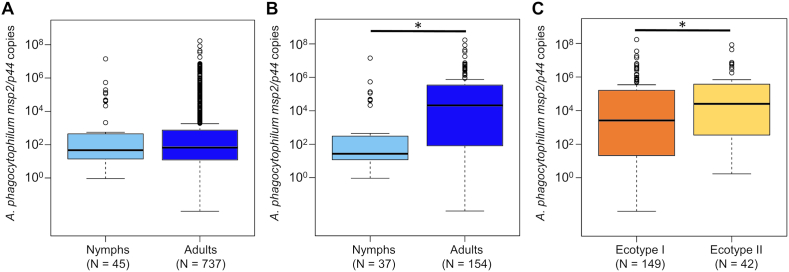
*Anaplasma phygocytophilum msp2/p44* copy numbers among all qPCR-positive ticks according to tick developmental stage (**A**), and in the data subset of successfully sequenced samples according to developmental stage (**B**) and *A. phagocytophilum* ecotype (**C**). Significant differences indicated by an asterisk were determined in a linear mixed model. The full model differed significantly from the null model containing only the random factor (*χ*^2^ = 13.9, *df* = 2, *P <* 0.001).

The ecotype distribution was similar between tick developmental stages, with Ecotype I identified in 75.0% (33/44) and 75.9% (123/162) and Ecotype II in 25.0% (11/44) and 24.1% (39/162) of nymphs and adults, respectively (Fisher’s exact test, *P =* 1.000). In the overall dataset of ticks with determined copy numbers, *msp2/p44* copies did not differ significantly between nymphal (*n =* 45) and adult *I. ricinus* (*n =* 737) (Wilcoxon rank sum test, *W* = 16662, *P =* 0.957). However, when considering only the samples which were successfully sequenced, copy numbers differed significantly according to both tick stage and ecotype. While copy numbers were higher in adult (*n* = 154) than nymphal (*n* = 37) ticks in this data subset (LMM, estimate [*Est*.] = 3.2, standard error [*SE*] = 0.96, *t* = 3.3, *P* = 0.001), they were also significantly higher in samples determined as Ecotype II (*n* = 42) compared to those assigned to Ecotype I (*n =* 149) (LMM, *Est*. = 1.6, *SE* = 0.82, *t =* 2.0, *P =* 0.049; Fig. 1).

### 3.3 Urban questing ticks

**Fig. 2:**
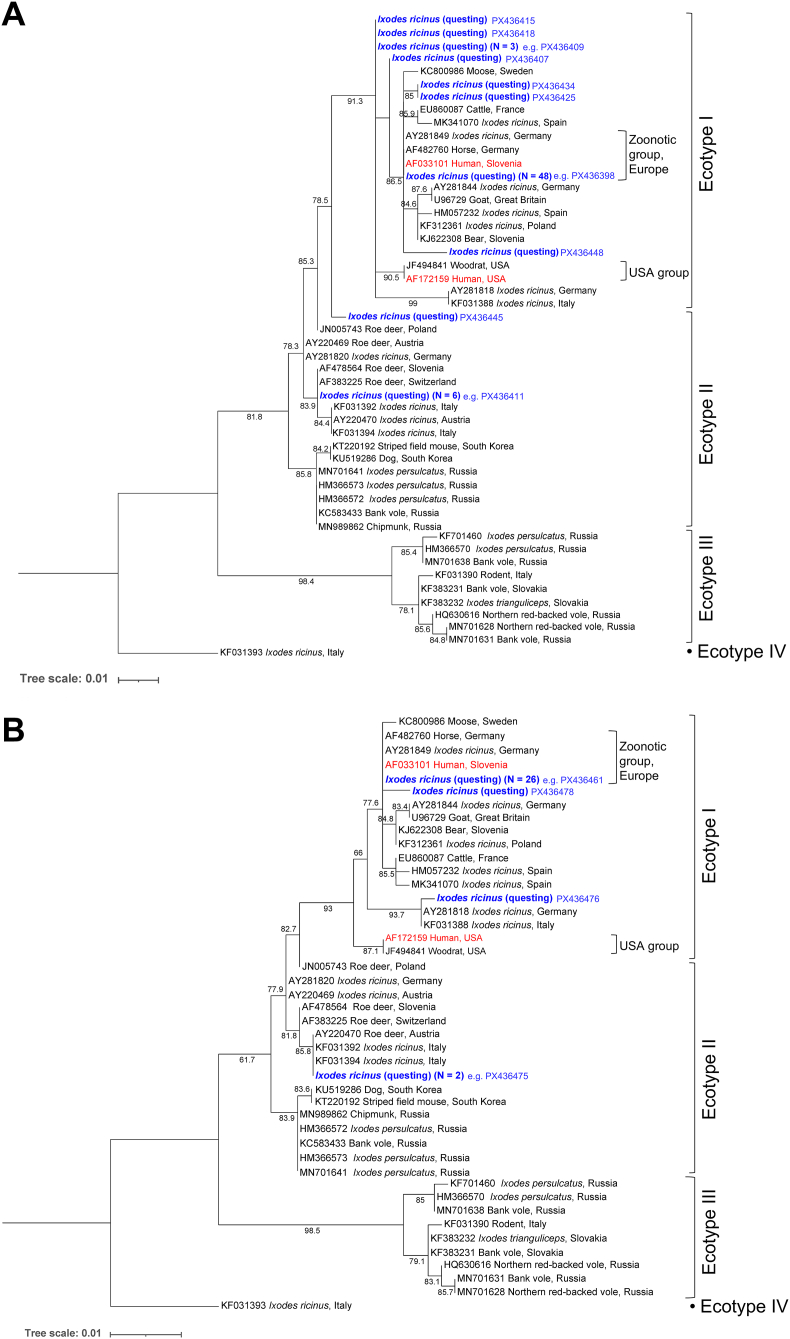
Maximum likelihood phylogenetic trees based on *A. phagocytophilum groEL* sequences (291 bp) generated in the present study (*blue*) from questing *I. ricinus* ticks collected in the city of Hannover, Germany, during 2015 (**A**) and 2020 (**B**), and reference sequences from NCBI GenBank. Sequences derived from human isolates are shown in *red*. Ultrafast bootstrap support values > 50% are indicated. Tree scale is in substitutions/site. The trees were constructed under a Tamura-Nei nucleotide substitution model with equal base frequencies and gamma-distributed rate heterogeneity (TNe+G4) (**A**), and Kimura 2-parameter model with gamma-distributed rate heterogeneity (K2P+G4) (**B**), respectively. Ecotype and group designations are given according to Rar et al. (2021).

In total, 94 of 125 *A. phagocytophilum*-positive questing ticks were successfully sequenced (Table 1). Of the 75 available *A. phagocytophilum*-positive ticks collected in the city of Hannover in 2015, 64 yielded a *groEL* sequence, while 30 of 50 samples from 2020 could be successfully sequenced (Table 1). Among these, 14 and four unique *groEL* sequences were represented, respectively (Fig. 2). There was no significant difference in ecotype distribution between the two study years (Fisher’s exact test, *P =* 0.714). In 2015, 89.1% of samples yielded sequences clustering within Ecotype I, and 10.9% within Ecotype II (Table 1). In 2020, the ecotype distribution was 93.3% Ecotype I and 6.7% Ecotype II. Considering both years together, the proportion of Ecotype I was lower in nymphs (82.4%, 28/34) than in adults (95.0%, 57/60), with 17.6% (6/34) and 5.0% (3/60) assigned to Ecotype II, respectively. However, this difference was not statistically significant (Fisher’s exact test, *P =* 0.067).

**Table 1:** *Anaplasma phagocytophilum* prevalence and ecotype distribution in *Ixodes ricinus* ticks from Germany.

Tick origin	Prevalence	No. subjected to *groEL* PCR	No. successfully sequenced	Ecotype I (% of identified samples)	Zoonotic group within Ecotype I (% of identified samples)	Ecotype II (% of identified samples)
Questing (city of Hanover)						
Collection year 2015	3.8% (79/2100)^a^	75	64 (85.3%)	57 (89.1)	48 (75.0)	7 (10.9)
Collection year 2020	3.0% (63/2100)^b^	50	30 (60.0%)	28 (93.3)	26 (86.7)	2 (6.7)
Total both years	3.4% (142/4200)	125	94 (75.2%)	85 (90.4)	74 (78.7)	9 (9.6)
Detached from humans	3.2% (42/1333)	42	27 (64.3%)	15 (55.6)	11 (40.7)	12 (44.4)
Detached from dogs	19.0% (285/1500)^c^	266	57 (21.4%)	39 (68.4)	31 (54.4)	18 (31.6)
Detached from cats	30.9% (464/1500)^c^	377	28 (7.4%)	17 (60.7)	4 (14.3)	11 (39.3)

a Blazejak et al. (2017).

b Glass et al. (2022).

c Probst et al. (2024).

**Fig. 3:**
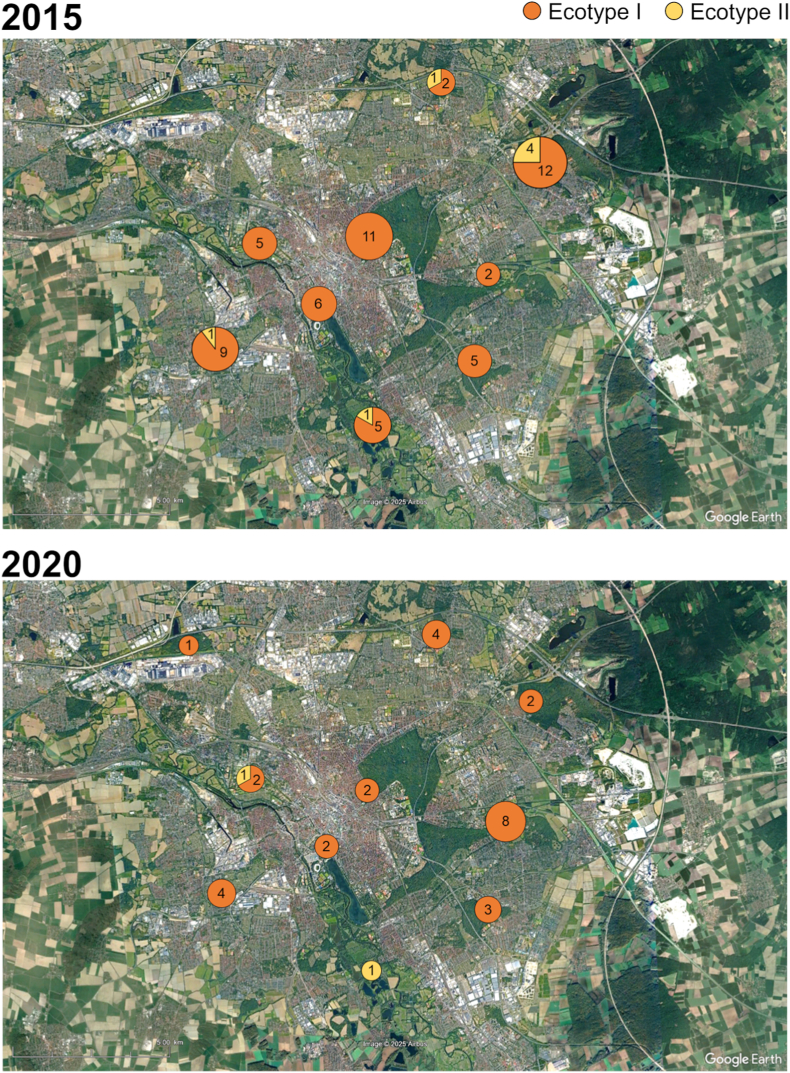
Distribution of *Anaplasma phagocytophilum* ecotypes in questing *I. ricinus* in 2015 and 2020 according to collection site in the city of Hannover, Germany. The number of successfully sequenced samples is given within the circles. Satellite image: Google Earth.

The majority of samples from both years (75.0% from 2015 and 86.7% from 2020) yielded sequences 100% identical to sequences of the “zoonotic group” within Ecotype I (e.g. GenBank: AF033101 and AF482760). The ecotype distribution at the ten different locations is visualized in Fig. 3, with Ecotype II occurring predominantly in peripheral parts of the city.

### 3.4 Ticks from humans

**Fig. 4:**
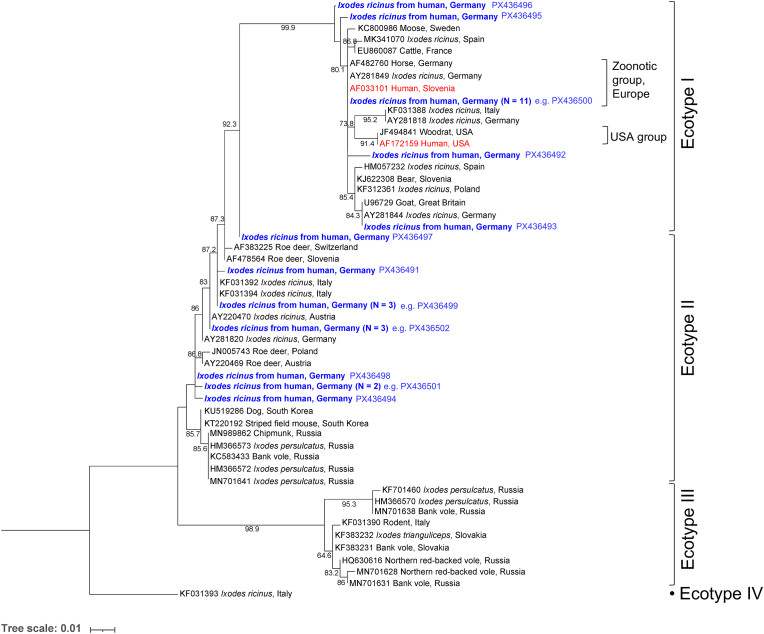
Maximum likelihood phylogenetic tree based on *A. phagocytophilum groEL* sequences (338 bp) generated in the present study (*blue*) from *I. ricinus* ticks detached from humans, and reference sequences from NCBI GenBank. Sequences derived from human isolates are shown in *red*. Ultrafast bootstrap support values > 50% are indicated. Tree scale is in substitutions/site. The nucleotide substitution model was a Tamura-Nei model with equal base frequencies and a proportion of invariant sites (TNe+I). Ecotype and group designations are given according to Rar et al. (2021).

Overall *A. phagocytophilum* detection frequency in *I. ricinus* from humans amounted to 3.2% (Table 1). In nymphs, the frequency was with 2.2% (16/726) significantly lower compared to adult ticks with 4.4% (25/565) (*χ*^2^-test, *χ*^2^ = 4.4, *df* = 1, *P* = 0.035). Further, one of 35 tested larvae (2.6%) and none of eight ticks with unidentified developmental stage were positive. Testing of 143 samples with 10 as well as 2 μl template by qPCR gave a consistent result with a detection frequency of 1.4% (2/143). Of the 42 qPCR-positive samples, 27 were successfully sequenced, yielding 12 unique *groEL* sequences (Fig. 4). The majority of these ticks originated from Germany, except for one tick each acquired in Poland and Denmark, as indicated by the sender. In total, 55.6% of samples contained sequences assigned to Ecotype I, and 44.4% to Ecotype II. In nymphs, the ecotype distribution was 50.0% (5/10) Ecotype I and II, respectively, and in adults 58.8% (10/17) Ecotype I and 41.2% (7/17) Ecotype II, without a significant difference (Fisher’s exact test, *P* = 0.706). Sequences 100% identical to those of the “zoonotic group” within Ecotype I were present in 40.7% of samples (Table 1).

### 3.5 Ticks from dogs and cats

**Fig. 5:**
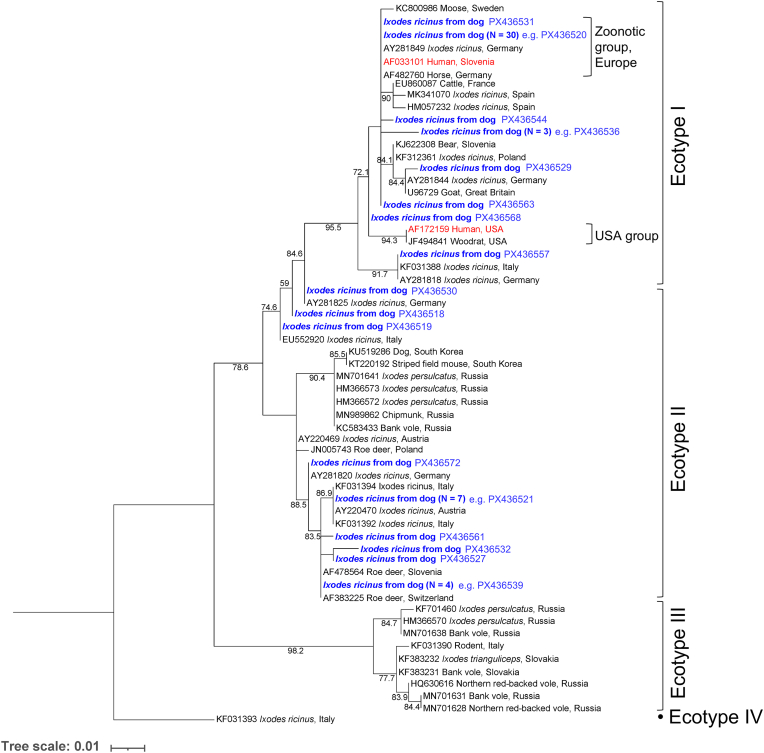
Maximum likelihood phylogenetic tree based on *A. phagocytophilum groEL* sequences (292 bp) generated in the present study (*blue*) from *I. ricinus* ticks detached from dogs, and reference sequences from NCBI GenBank. Sequences derived from human isolates are shown in *red*. Ultrafast bootstrap support values > 50% are indicated. Tree scale is in substitutions/site. The nucleotide substitution model was a Tamura-Nei model with equal base frequencies and gamma-distributed rate heterogeneity (TNe+G4). Ecotype and group designations are given according to Rar et al. (2021).

**Fig. 6:**
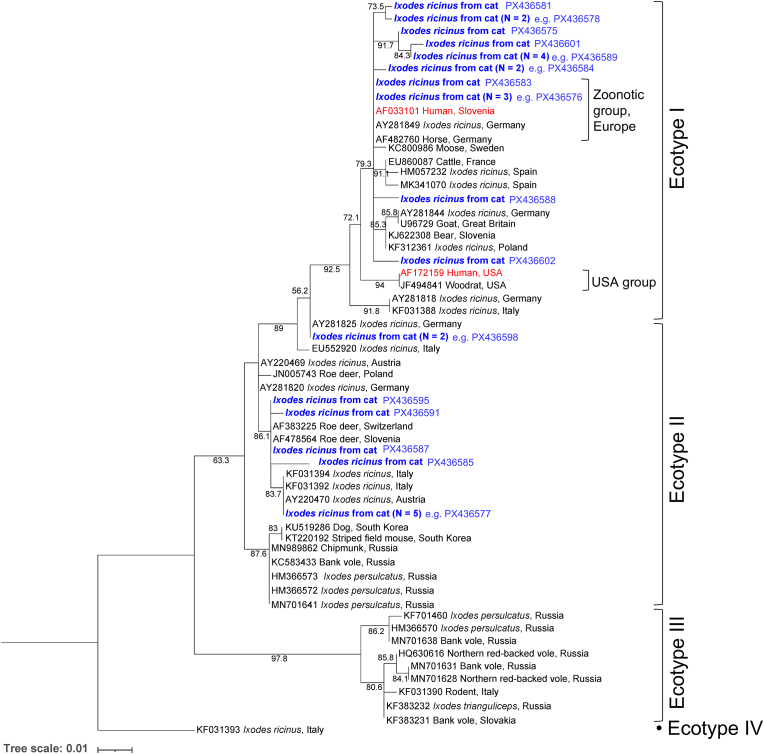
Maximum likelihood phylogenetic tree based on *A. phagocytophilum groEL* sequences (290 bp) generated in the present study (*blue*) from *I. ricinus* ticks detached from cats, and reference sequences from NCBI GenBank. Sequences derived from human isolates are shown in *red*. Ultrafast bootstrap support values > 50% are indicated. Tree scale is in substitutions/site. The nucleotide substitution model was a Tamura-Nei model with equal base frequencies and gamma-distributed rate heterogeneity (TNe+G4). Ecotype and group designations are given according to Rar et al. (2021).

Of the 266 female *I. ricinus* from dogs subjected to *groEL* PCR, 57 were successfully sequenced, yielding 17 unique sequences (Fig. 5). Ecotype I was present in 68.4% of samples, with the “zoonotic group” represented by 54.4%, and Ecotype II in 31.6% (Table 1). Of the 377 samples from cats, 28 were successfully sequenced, comprising 16 unique sequences (Fig. 6). The ecotype distribution was 60.7% Ecotype I, including 14.3% clustering with the “zoonotic group”, and 39.3% Ecotype II (Table 1).

Of the 57 successfully sequenced samples from dogs, 43 were from dogs with a rural residence and 11 from dogs with an urban residence, while no information was provided for the remaining three samples. The 28 sequenced samples from cats comprised 23 from cats with a rural residence and three from cats with an urban residence, with no information for the remaining two samples. Considering ticks from both host species together, no significant influence of the type of residence (GLM, *Est*. = −0.78, *SE* = 0.70, *z* = −1.1, *P* = 0.265) nor the host species (GLM, *Est*. = 0.16, *SE* = 0.50, *z* = 0.33, *P* = 0.745) on ecotype distribution was found.

### 3.6 Differences in ecotype distribution between questing and host-attached ticks

No significant differences in ecotype distribution were noted between ticks from humans, dogs and cats (Fisher’s exact tests, Bonferroni-Holm-adjusted *P* = 1.000). However, the proportion of Ecotype I was significantly lower in human-, dog- and cat-associated ticks compared to the urban questing ticks (Fisher’s exact tests, Bonferroni-Holm-adjusted *P <* 0.001, *P =* 0.004 and *P =* 0.003, respectively).

## 4 Discussion

The aim of this study was to elucidate the *A. phagocytophilum* ecotype distribution, and in particular the frequency of potentially zoonotic strains in questing and host-derived *I. ricinus* from Germany. Although multiple markers can be used for genotyping *A. phagocytophilum*, the different methods usually show a high level of concordance (Rar et al., 2021; Kruppenbacher et al., 2023), and the *groEL* gene was chosen here for comparison with most previous studies (e.g. Di Domenico et al., 2016; Hamšíková et al., 2019; Jaarsma et al., 2019; Gandy et al., 2022; Lesiczka et al., 2023). Although the obtained sequences were rather short (290–338 bp), they nevertheless allowed an unambiguous ecotype classification. Moreover, reference sequences previously identified as belonging to the “zoonotic subgroup” within Ecotype I (Rar et al., 2021) also clustered together unambiguously in the phylogenetic trees generated in the present study. Lesiczka et al. (2025) obtained a similar result based on 366 bp of the *groEL* gene. Nevertheless, longer sequences may allow more resolution overall, so other clusters within the phylogenetic trees presented here should be interpreted with caution.

In line with previous investigations (Hamšíková et al., 2019; Lesiczka et al., 2021; Gandy et al., 2022), only *A. phagocytophilum* Ecotypes I and II were detected in *I. ricinus*, with an overall predominance of Ecotype I. The proportion of Ecotype I was particularly high in the questing urban ticks in this study, with only one out of ten ticks from the city of Hanover harbouring Ecotype II. A similar ecotype distribution has been detected in questing ticks from the UK (Gandy et al., 2022) and from Norway (Stigum et al., 2019), while a higher Ecotype II proportion of almost 30% was determined in questing ticks from different habitats in the Czech Republic, Slovakia and the southern German federal state Bavaria (Hamšíková et al., 2019). Previous studies have shown an association between the presence of roe deer, which are regarded as the main reservoir of Ecotype II, and the frequency of this ecotype in questing *I. ricinus* (Hamšíková et al., 2019; Stigum et al., 2019). In the city of Hanover, Ecotype II was only identified at a few locations, primarily in peripheral parts of the city, where roe deer are more likely to be present than at inner-urban locations, as indicated by wildlife camera surveys (unpublished data). Moreover, almost 80% of *groEL* sequences from questing ticks clustered with the “zoonotic group” within Ecotype I. In contrast, a Europe-wide analysis of *groEL* sequences present in GenBank indicated that the zoonotic variant was far less abundant in questing *I. ricinus* than non-zoonotic variants of Ecotype I (Lesiczka et al., 2025). However, the authors caution that the prevalence of the zoonotic variant may show geographical variation, e.g. depending on host community. The zoonotic group does not only comprise isolates known to cause disease in humans, dogs, cats and horses, but additionally almost all isolates from wild boar, red foxes and hedgehogs (Rar et al., 2021). Especially hedgehogs are suspected to be key reservoirs for tick-borne pathogens in urban areas, showing a high prevalence of *A. phagocytophilum* infections (Lesiczka et al., 2021; Springer et al., 2024), and may contribute to the high proportion of potentially zoonotic strains in the urban ticks of the present study.

Moreover, host-derived ticks were investigated in the present study. As neither humans nor dogs and cats are regarded as reservoirs for *A. phagocytophilum*, the presence of the pathogen most likely reflected the tick’s prior infection status, not a recent uptake *via* the blood meal. This is also corroborated by a previous study, showing that in cases of multiple infestations, most dogs and cats carried only a single infected tick, and that the probability of infection decreased rather than increased with increasing tick engorgement duration (Probst et al., 2024). The proportion of Ecotype I, and hence of the “zoonotic group”, was significantly lower in the host-derived ticks, including those from humans, compared to questing ticks. Only 55.6% of ticks detached from humans yielded sequences clustering within Ecotype I, and 40.7% with the “zoonotic group”, compared to 90.4% and 78.7%, respectively, in the urban questing ticks. Considering the overall *A. phagocytophilum* prevalence of 3.2% in human-derived ticks, approximately 1.3% of ticks feeding on humans pose a risk of HGA. In comparison, the prevalence of the North American *A. phagocytophilum* variant Ap-ha, which is considered human-pathogenic, ranged from 0 to 8.9% in adult and 0 to 5.1% in nymphal *Ixodes scapularis* from different states of the USA (Hojgaard et al., 2024). Thus, a lower prevalence of zoonotic strains may indeed contribute to the lower HGA incidence in Europe compared to North America, in addition to potential differences in pathogenicity between the European and American zoonotic strains (Aardema, 2023).

The difference in ecotype distribution between questing and host-derived ticks is probably related to their geographical origin. Whereas questing ticks were collected from ten defined areas within the city of Hanover, feeding ticks were derived from all over Germany and were probably acquired in a range of different landscapes, including rural areas where roe deer are likely present in greater numbers than in urban areas. Thus, the risk of granulocytic anaplasmosis may differ geographically and/or between urban and rural areas, depending on the reservoir host fauna. Unfortunately, not all senders provided information on the area where the tick was probably acquired, so that a comparison of ecotypes between human-detached ticks from rural and urban areas was not possible.

Amplification and sequencing success for *I. ricinus* females from dogs and cats amounted to only 21.4% and 7.4%, respectively, compared to more than 60% for questing ticks and ticks detached from humans. This can be attributed to the generally low *A. phagocytophilum* copy numbers in ticks from dogs and cats (Probst et al., 2024). Most samples for which no sequence could be generated yielded no visible amplicon band. The fact that the *msp2/p44* targeted by the qPCR is a multi-copy gene (Courtney et al., 2004) probably contributed to the difference in sensitivity between qPCR and conventional PCR in the present study. Additionally, in approximately 10% of cases, amplicons were sent for sequencing, but yielded either no or a low-quality sequence, or a non-*A. phagocytophilum* sequence (mostly *Pseudomonas* spp.), indicating that the presence of other bacteria may interfere with the assay. In a few cases, mixed infections with different *A. phagocytophilum* ecotypes may have prevented a high-quality sequencing result. Nevertheless, 57 samples from dogs and 28 samples from cats were successfully sequenced, with 68.4% and 60.7% identified as Ecotype I and 31.6% and 39.3% as Ecotype II, respectively. Approximately two-thirds of these ticks were collected from pets with a rural residence, which may explain the higher proportion of Ecotype II compared to the urban questing ticks. However, when taking host species into account, no statistically significant difference in ecotype distribution according to the type of residence was found. Since almost 90% of the sequenced samples from cats were from rural areas, it remains unclear whether the high Ecotype II proportion among these ticks is related to the host species or to the area of residence.

Sequences clustering with the “zoonotic group” within Ecotype I were detected in 54.4% of ticks from dogs. This group also contains isolates from clinical canine granulocytic anaplasmosis (CGA) cases (Dondi et al., 2014; Rar et al., 2021). Given the high *A. phagocytophilum* detection frequency of 19.0% in ticks from dogs (Probst et al., 2024), this indicates that approximately one out of ten *I. ricinus* females parasitising dogs may pose a risk of CGA. In contrast, only 14.3% of the *I. ricinus* females from cats yielded sequences clustering with the “zoonotic group”. In addition to the possible influence of a rural *vs* urban residence discussed above, this difference may be related to the animals’ behaviour. Cats usually roam independently, while dogs are mostly accompanied by their owners. This may explain the similar proportion of potentially zoonotic isolates found in ticks from humans and dogs, while cats may come into contact with ticks that previously fed on a larger variety of hosts. Feline granulocytic anaplasmosis (FGA) is increasingly diagnosed (Schäfer et al., 2022) but still considered rather rare, although the same strains cause clinical disease in cats as in humans and dogs (Kruppenbacher et al., 2023). Aside from under-diagnosis as suggested by Kruppenbacher et al. (2023), a lower level of exposure with pathogenic strains may contribute to the low FGA incidence, despite the fact that every third *I. ricinus* female from cats was *A. phagocytophilum*-positive (Probst et al., 2024). Nevertheless, the low proportion of isolates clustering with the zoonotic group in ticks from cats was somewhat surprising, given the fact that, next to *I. ricinus*, the hedgehog tick *Ixodes hexagonus* was collected quite frequently from cats (Probst et al., 2023). This indicates a high degree of habitat overlap with hedgehogs, which mostly carry *A. phagocytophilum* strains from the zoonotic group (Rar et al., 2021).

Interestingly, *A. phagocytophilum msp2/p44* copy numbers were significantly higher in samples assigned to Ecotype II compared to Ecotype I, in addition to significantly higher copy numbers in adult than nymphal ticks. To the authors’ knowledge, this is the first study investigating *A. phagocytophilum* copy numbers in relation to ecotype. These ecotype differences in copy numbers might indicate either a difference in tick infection intensity, or a difference in the number of *msp2/p44* gene paralogs between different ecotypes, as this is a multicopy gene (Murphy et al., 1998; Caspersen et al., 2002). If Ecotype II strains indeed show a higher infection intensity in ticks, this might have influenced the detected ecotype distribution, with a potential underrepresentation of Ecotype I, due to the limited sensitivity of the conventional *groEL* PCR. Therefore, the proportion of Ecotype I and of potentially zoonotic isolates might in fact be even higher.

## 5 Conclusions

Among *A. phagocytophilum*-positive questing *I. ricinus* from the urban area of Hanover, almost 80% harboured isolates clustering with the “zoonotic group”, potentially pathogenic for humans and their pets. This proportion was lower in specimens removed from humans, dogs and cats countrywide, indicating geographical and/or rural-urban differences in exposure risk towards pathogenic strains. Nevertheless, every second *A. phagocytophilum*-positive tick from dogs and almost every second tick from humans carried potentially pathogenic isolates, highlighting the importance of preventive measures to avoid tick infestation. Ticks from cats harboured the lowest proportion of potentially pathogenic isolates, although it remains unclear whether this was driven by the animal species or their mostly rural origin. Therefore, further studies are necessary to shed more light on the risk of feline granulocytic anaplasmosis.

## Ethical approval

Not applicable.

## CRediT authorship contribution statement

**Andrea Springer:** Investigation, Formal analysis, Visualization, Writing - original draft. **Daniela Angulo Mora:** Investigation, Writing - review & editing. **Daniela Jordan:** Investigation, Writing - review & editing. **Christina Strube:** Conceptualization, Project administration, Supervision, Writing - review & editing.

## Funding

This research did not receive any specific grant from funding agencies in the public, commercial, or not-for-profit sectors.

## Declaration of competing interests

The authors declare the following financial interests/personal relationships which may be considered as potential competing interests: Christina Strube has repeatedly lectured for and acted as a consultant for diagnostic and (veterinary) pharmaceutical companies and has previous and ongoing research collaborations with various diagnostic and (veterinary) pharmaceutical companies. The other authors declare that they have no known competing financial interests or personal relationships that could have appeared to influence the work reported in this paper.

## Data Availability

The data supporting the conclusions of this article are included within the article. The sequences generated during this study were submitted to NCBI GenBank under the accession nos. PX436397-PX436602.
